# Epidemiological Investigation on the Clinical Status of the Developmental Dyslexia and ADHD Comorbidity among School-Age Children in Pakistan

**DOI:** 10.4236/ojepi.2025.153033

**Published:** 2025-08-01

**Authors:** Shujjah Haider, Tanmoy Mondal, Christopher A. Loffredo, Brent Korba, Irum Nawaz, Maleeha Azam, Tapas Sur, Somiranjan Ghosh

**Affiliations:** 1 Department of Biosciences, COMSATS University Islamabad, Islamabad, Pakistan; 2 Department of Biology, Howard University, Washington DC, USA; 3 Department of Oncology, Georgetown University, Washington, DC, USA; 4 Department of Microbiology & Immunology, Georgetown University, Washington DC, USA; 5 Faculty of Rehabilitation and Allied Health Sciences, Riphah International University, Islamabad, Pakistan; 6 Department of Pediatrics and Child Health, College of Medicine, Howard University, Washington DC, USA

**Keywords:** Neurodevelopmental Disorder, Dyslexia, ADHD, Biochemical Parameters, Sociodemographic Factors

## Abstract

Developmental Dyslexia (DD) and Attention-Deficit/Hyperactivity Disorder (ADHD) are neurodevelopmental disorders affecting children’s learning abilities worldwide, but limited research exists in Pakistan. We tried to identify and confirm ADHD and DD in Pakistani school children and their sociodemographic associations. We conducted a case-control study, examining an extensive cohort of children (n = 1260), aged 5 - 19 years from special education program schools, rehabilitation centers, and pediatric clinics between 2022 and 2023. Standardized psychological evaluations were conducted to confirm cases, and clinical, biochemical, demographic, and family history data were collected. About 288 children were identified and diagnosed with ADHD or DD, compared to 27 controls. In our child cohort males dominate the distribution (65%) compared to females (35%) compared to 56% and 44% representation of males and females respectively in controls. Our findings revealed an extensive gender difference within the cases and a significant difference (p-value = 0.05) with Hydroxy D3 levels compared to the control group. Our observations are consistent with previous studies, showing a lack of associations with sociodemographic and blood biochemical levels, suggesting other factors may influence the development of these disorders.

## 1. Introduction

Developmental Dyslexia (DD) and Attention-Deficit/Hyperactivity Disorder (ADHD) are neurodevelopmental disorders characterized by distinct and overlapping symptoms that include inattention, hyperactivity, and impulsivity in ADHD, and difficulties in reading and language processing in DD [1]. Both conditions involve genetic, environmental, and neurological factors contributing to their development, and they can persist from childhood into adulthood [2]. Commonly, DD patients exhibit delayed spoken language, letter transposition in words, slow reading, and confusion with direction, poor comprehension, and limited vocabulary [3]. Furthermore, these conditions may also be co-morbid with depressive, bipolar, schizophrenia, and anxiety disorders [4]. Research indicates that individuals can have both disorders concurrently, which can complicate their learning ability and behavioral patterns, impairing their conditions [5]. School-attending children diagnosed with reading disability and hyperactivity demonstrate poor academic attainments and are at an elevated risk of behavioral challenges [6] [7]. Several studies indicate that clinical (biochemical) conditions (e.g., low ferritin and Vitamin D) are associated with such complex neurobehavioral disorders, but this research has not been conclusive [8]-[10]. The worldwide prevalence of DD is 3% to 7%, while ADHD is 5%, and they have a male-to-female ratio of 3:1 [11] [12]. World Health Organization (WHO) data shows that 10% of the global population has a learning disability [7]. Estimates show that 66.3% of diagnosed children and adolescents take medication for the disorder, among whom 4.8% of children are in the age group of 4 - 17 years [6] [13] [14]. In Pakistan, the prevalence of learning disabilities has been estimated from 15% to 20% [15]. This suggests that currently more than 12 million children in Pakistan require special educational programs for these disorders [15] [16].

In the present study, we aimed to identify and confirm ADHD and DD among a broad sample of Pakistani school children. We also explored the associations between these conditions and various risk factors, including gender, family history, and mode of delivery, consanguinity, area of residence, family education, profession, and biochemical and clinical parameters in the affected children, comparing them with a control group.

## 2. Methods

### 2.1. Ethical Statement

This study was approved by the Ethical Review Board (ERB) of (CUI), Islamabad, Pakistan (CUI/BIO/ERB/2022/11), on December 19, 2022, and the Howard University Institutional Review Board (IRB-2023-0810) on May 30, 2023, the latter institution approving the transfer of biological specimens to the US for molecular assays. Informed written consents were obtained from the parents or legal guardians prior to enrolment in the study. This research was conducted ethically in accordance with the World Medical Association Declaration of Helsinki.

### 2.2. Study Design and Participants

Children aged 5 to 19 years were screened from special education program schools including children’s rehabilitation centers (n = 750) and pediatric neuropsychiatric clinics (n = 510) in four major cities: Chakwal, Rawalpindi, Islamabad, and Lahore. For the control group recruitment participants were randomly selected from a regular school in the same area. Initially, a total of 110 children aged between 5 and 19 years were enrolled, and their parents expressed a willingness to participate in the study. However, ultimately, only 27 participants (children) agreed to undergo the psychological evaluation and blood draw. Children with major neurological disorders, impairment in hearing or vision, and those with severe medical conditions were excluded. Medical records were obtained from all recruited participants, including gender, age, residence area, family history, consanguinity, mode of delivery, parents’ education level, and parents’ smoking.

### 2.3. Psychological Evaluation

To diagnose ADHD and DD, we measured the neuroadaptive profile of each child. It was evaluated by standardized psychological evaluation methods following the criteria of DSM-V [17]. The assessments were conducted in accordance with internationally recognized standard protocols, which included the use of available Urdu-translated questionnaires and tests [15] [18]. Nonverbal assessment was conducted using the Standard Progressive Matrices (SPM) [19]. To evaluate learning ability, problem-solving skills, comprehension, reasoning, judgment, and knowledge retention, the Urdu version of the Slosson intelligence test (SIT R3) was utilized [20] [21]. Reading-writing ability, quantitative knowledge, and cognitive processing speed were measured using the Woodcock-Johnson Tests of Cognitive Abilities (WJ-IV) and the Bangor Dyslexia test [22]-[24]. Additionally, for a comprehensive evaluation of potential behavioral markers of ADHD in children aged 6 - 18 years, the Conners Parent Rating Scale-Long Version (CPRS-R: L) was used. This scale assesses various aspects comprising inattention, hyperactivity/impulsivity, learning problems, executive functioning, and aggression [25] [26].

### 2.4. Clinical Blood Tests

Clinical biochemistry parameters were retrieved from the participants’ medical records to investigate potential associations. These parameters include blood profile, non-fasting glucose, liver function tests, renal function tests, and Vitamin D3 (VIT-D3) levels. The tests were conducted using the Cobas 6000 Modular System from Roche Diagnostics, Germany, at the Islamabad Diagnostic Laboratory (IDC) in Pakistan.

### 2.5. Statistical Analysis

We performed two types of statistical analyses to compare cases to controls. Firstly, independent sample t-tests were conducted for continuous variables, including age, serum glucose, WBCs, RBCs, hemoglobin, PVC, MCV, MCH, platelets, polymorph, lymphocytes, Monocytes, serum Bilirubin total, serum ASAT, serum alkaline phosphate, serum creatinine, serum sodium, serum potassium, serum chloride, and Hydroxy vitamin D3 level [27]. Chi-square tests were computed for categorical variables such as gender, pregnancy (mode of delivery), area of residence, family education, family profession, and marriage [28]. All values were presented using percentages or means and standard deviations (±SD). Statistical significance was set at *p* ≤ 0.05. All statistical analyses were performed using SPSS, version 29.0.

## 3. Results

### 3.1. Participant’s Characteristics

Out of a total of 1260 participants, we diagnosed 288 with ADHD and DD (cases) based on scores obtained from our psychological test battery. Based on the psychological evaluation tests, we identified three case subgroups: DD (n = 192), ADHD (n = 20), and DD co-morbid with ADHD (n = 76). A total of 27 children tested normal on all parameters (controls). The average age of the study population (all cases and controls combined) was 13.67 ± 3.03, comprising 187 males (64%) and 101 females (36%) from the case group, and 15 males (56%) and 12 females (44%) in the control group. The mean age in the control and case groups was 11.89 ± 3.67 and 13.84 ± 2.92 years, respectively. In the case group, 20% of participants were aged 6 - 11 years, while 1% were aged 3 - 5 years, with most (71%) falling within the 12 - 17 age range (Table 1). In the case group, 33% were involved in cesarean delivery (C-section), and 72% of families of participants reported consanguineous marriage (marriage within the same family) (Table 1). We observed no differences between cases and controls based on area of residence, family education, and family profession (Table 1).

### 3.2. Psychological Evaluation Results

The mean scores of the Standard Progressive Matrices (SPM), Slosson Intelligence Test (SIT R3 adopted Urdu version), and Woodcock-Johnson IV Tests of Achievement (WJ IV Dyslexia), as well as the Bangor Dyslexia Test and Conners Comprehensive-Behavior Rating Scales for DD and ADHD exhibited statistically significant differences from the control group (p values < 0.005), as detailed in Table 2.

### 3.3. Clinical Blood Test Results

**Figure 1.**
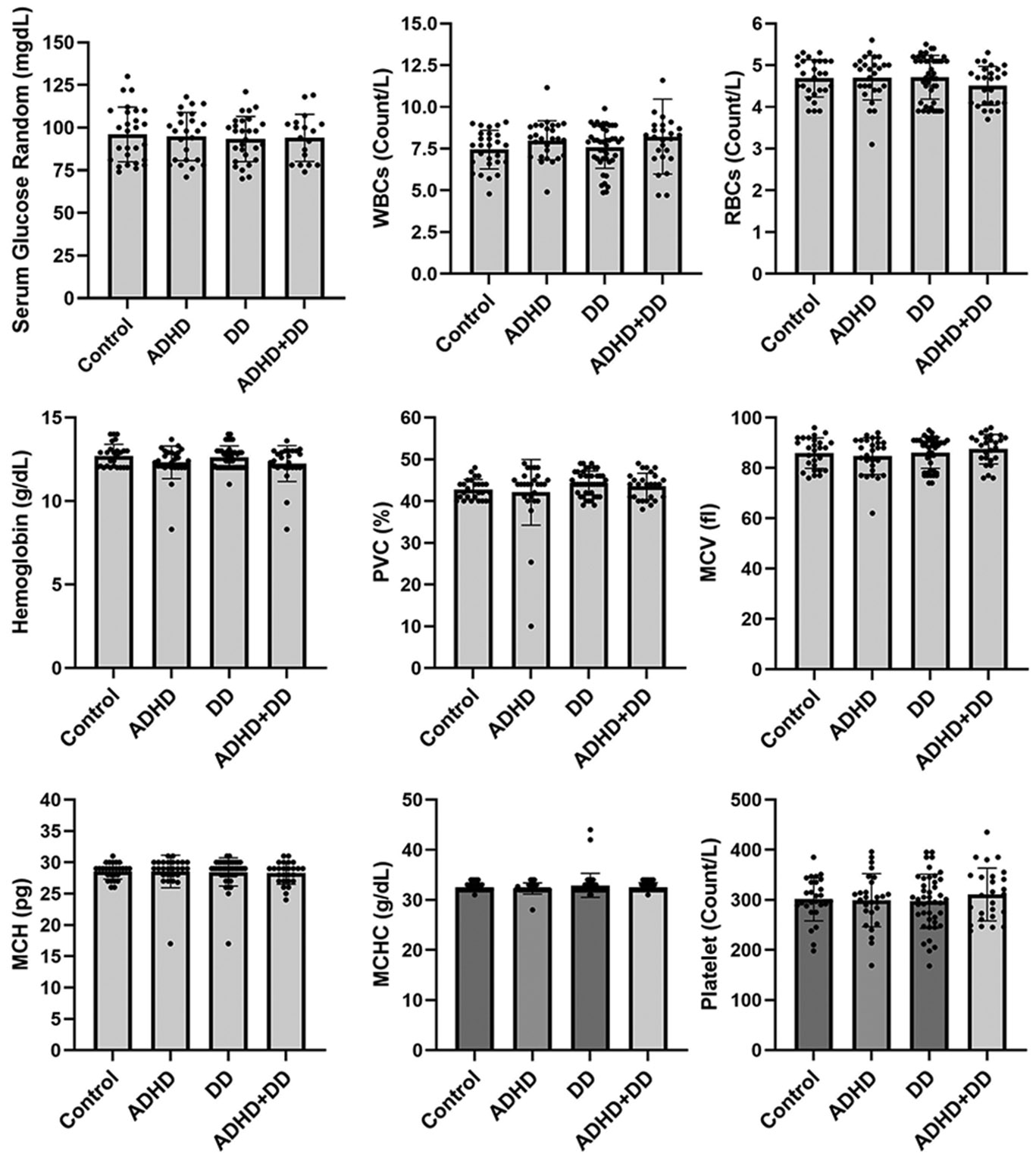
Blood biochemical parameters, such as serum glucose random, WBCs, RBCs, hemoglobin, PVC, MCV, MCH, MCHC, and platelet levels, were assessed among the control group, ADHD group, DD group, and the ADHD comorbid with DD group.

**Figure 2.**
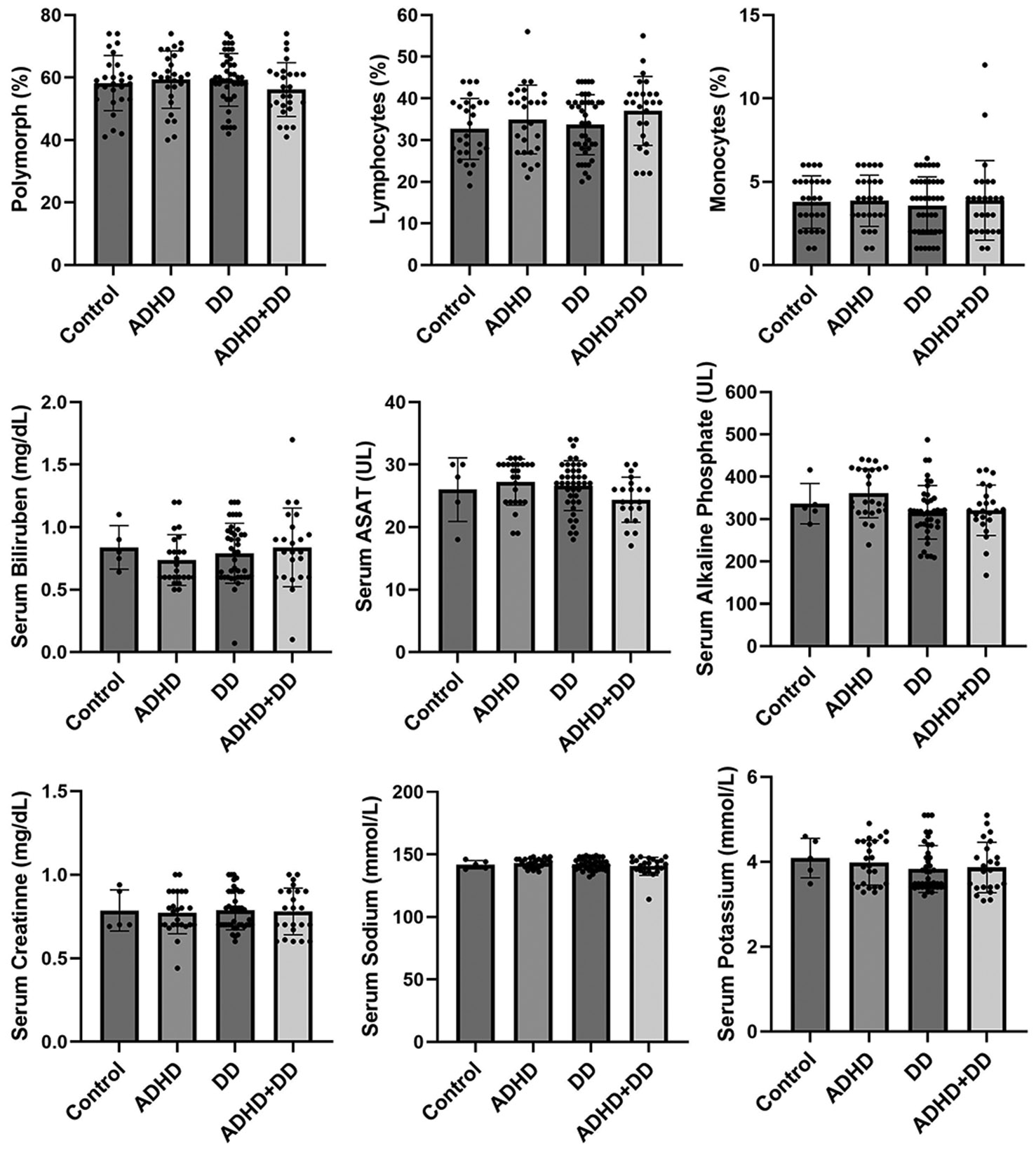
Blood biochemical parameters, such as Polymorph, Lymphocytes, Monocytes, Serum Bilirubin, Serum ASAT, Serum alkaline phosphate, Serum creatinine, Serum sodium, and Serum potassium levels, were assessed among the control group, ADHD group, DD group, and the ADHD co-morbid with DD group.

The case group exhibited a marginal difference (p value 0.05) in the mean vitamin D3 level in the case group (20.06 ± 11.97 ng/ml) compared to the control group (31.40 ± 5.01 ng/ml) (Table 3), with cases testing below the normal range of 30 - 100 ng/ml. All additional hematological tests were within normal ranges across the study participants, including Random Glucose level, WBCs, RBCs, Hemoglobin, PVC, MCV, MCH, MCHC, Platelets, Polymorph, Lymphocytes, and Monocytes. Similarly, liver function was within normal ranges, including Serum Bilirubin, Serum ASAT, and Serum alkaline Phosphate. Further renal function tests were also recorded as normal, including Serum creatinine, Serum Sodium, Serum Potassium, and Serum Chloride (Table 3). We did not observe any significant differences in clinical pathology parameters between the control group and any individual disease subgroup (Figure 1 and Figure 2).

## 4. Discussion

DD and ADHD are neurodevelopmental disorders that can co-occur in individuals. Both conditions have distinct characteristics, but they often share some overlapping features [3] [12]. We followed the international standard psychological evaluation batteries to diagnose and differentiate between study groups, including DD, ADHD, DD co-morbid with ADHD, and healthy controls. Dyslexia tests and the Conners Parent Rating Scale-Long Version (CPRS-R: L) for behavioral markers were conducted to differentiate DD and ADHD phenotypes in all study groups. The validity and application of all these tools align with previously conducted studies in the children population [15] [29]. Our psychological assessments provided a comprehensive evaluation of participants’ cognitive and behavioral profiles, revealing significant heterogeneity in their abilities and potential challenges. Notably, a significant number of participants demonstrated above-average performance in nonverbal reasoning and problem-solving skills, indicating a normal range of intellectual ability with strong cognitive capabilities. Despite these strengths, many participants exhibited dyslexic features and ADHD characteristics, as highlighted by the results of the Bangor Dyslexia Test and the Conners Comprehensive Behavior Test (Table 2). The presence of these dyslexic features raises concerns about potential impacts on reading and language processing within this population [30]. Similarly, data obtained from the Conners Comprehensive Behavior Test indicated a high prevalence of ADHD-related symptoms, particularly hyperactivity and impulsivity, among participants. These behavioral characteristics were most pronounced in individuals with co-occurring DD and ADHD. We observed that DD-categorized participants were in the majority, whereas DD co-morbid with ADHD was also high compared to the ADHD group. In overall our observation indicated that despite identifying ADHD or DD categorically through psychological tests, the participating children did not exhibit intellectual impairment. It is important to note that, as per the current psychological testing guidelines, these test scores provide valuable information; however, they should be interpreted in conjunction with other clinical assessments and observations to form a comprehensive understanding of an individual’s cognitive and psychological profile [31]. Other research studies have indicated that children with ADHD tend to exhibit notably lower serum levels of certain vitamins (specifically Vitamin D3, B12, and B6) and higher levels of saturated fatty acids compared to their peers [32] [33]. However, upon reviewing the overall medical records of the affected individuals in our study, no significant changes in their blood biochemical factors were detected, except for a significant insufficiency in vitamin D3 levels. Vitamin D receptors are widely distributed in the neuronal cells of the substantia nigra, hippocampus, hypothalamus, prefrontal cortex, and cingulated gyrus as these regions are significantly well studied in the pathogenesis of ADHD and DD [34] [35]. Furthermore, Vitamin D is essential to produce dopamine and norepinephrine neurotransmitters, which significantly alleviate severe symptoms of ADHD [36]. Our investigation into Vitamin D3 deficiency aligns with previously reported findings from numerous studies [34] [36].

In our study, 65% of male and 35% of female children with DD and ADHD were identified. The proportion of male participants was high, which is consistent with many other studies [37] [38]. Our data suggests a high percentage (70%) of reported cases of DD and ADHD, mostly within the 12- to 17-year age group among the total research participants, which is also consistent with previous findings in other populations [13]. Cases of female children with these disorders are typically much lower than in males, particularly in rural areas of Pakistan, as it is hard to include female participants due to lack of awareness and the social structures, which may account for fewer female subjects in the current study. Specific prenatal and perinatal factors, including low birth weight, prematurity, and mode of delivery (C-sections), play a major contributing role in developmental delay in children [39]. The reported cesarean section rate was high at 32% among the study participants, consistent with other studies. In our cohort, consanguinity rates were approximately 71% while previous studies also revealed that a higher rate of consanguineous marriage is significantly associated with neurodevelopmental disorders in Asian populations [7]. Many studies reported that the presence of a common susceptibility locus (AP4M1 p.E193K) in parents increases the risk of neuro-developmental disorders, including ADHD, schizophrenia, bipolar disorder, autism, and major depressive disorder in their progeny, but we had no data on this locus in our study [9]. Our study provides preliminary insights into ADHD and D) in major cities of Pakistan, highlighting their prevalence and clinical status. Given the lack of awareness, this work aims to address misconceptions and promote early interventions in families, schools, and communities. By contributing to the global database, our study may offer a model for diagnostic practices and socio-demographic analyses in low-resource settings. Future studies should focus on longitudinal studies with more selective cohorts and comparative research on adult populations to explore early-life influences on outcomes and improve risk assessment and therapeutic strategies.

This study had several strengths, including the ability to examine a large case group using standardized psychological batteries alongside clinical and demographic information. However, there were also some limitations. The small sample size of controls may have constrained the statistical significance of case-control comparisons. Furthermore, our study only included four cities in Pakistan, which limits the generalizability of our findings to the broader population.

## 5. Conclusion

The study identified and confirmed ADHD and DD among Pakistani school children using standard psychological batteries. The study highlighted a substantial gender difference and a lack of significant association with any sociodemographic characteristics and blood biochemical characteristics. Future studies should focus on genetic and molecular factors, to better understand the ADHD and DD conditions and correlates in Pakistani children.

## Figures and Tables

**Table 1.** Participants’ characteristics and socio-demographic status.

Demographic details	Control n (%)	Cases n (%)	*p*-value
Gender			0.33
Male	15 (56)	187 (65)	
Female	12 (44)	101 (35)	
Age			0.21
3 - 5	0 (0)	1 (1)	
6 - 11	11 (41)	58 (20)	
12 - 17	16 (59)	204 (71)	
18 or over	0 (0)	25 (8)	
Age on set			
3 - 5	-	147 (51)	
6 - 11	-	141 (49)	
Pregnancy (Mode of delivery)			0.45
Other	20 (74)	133 (67)	
C-Section	7 (26)	66 (33)	
Area of Residence			0.20
Rural	0 (0)	18 (6)	
Urban	27 (100)	252 (90)	
Industrial	0 (0)	12 (4)	
Family Education			0.24
High School or Below	12 (45)	44 (46)	
Collage BS/BA	9 (33)	41 (43)	
MS	5 (18)	10 (10)	
PHD	1 (4)	0 (0)	
Professional	0 (0)	1 (1)	
Family Profession			0.96
Gov/Private Employment	13 (48)	47 (49)	
Self Employed	9 (33)	34 (35)	
Farmer	5 (19)	16 (16)	
Marriage type			0.59
Non consanguineous	9 (33)	54 (28)	
Consanguineous	18 (67)	136 (72)	

**Table 2.** Psychological test results.

Clinical diagnostic details	Control n (%)	Cases n (%)	*p*-value
SPM in Category			<0.001
Intellectually Average	0 (0)	56 (19)	
Above the average	26 (96)	232 (81)	
Intellectually Superior	1 (4)	0 (0)	
Slosson Intelligent			<0.001
Borderline	7 (26)	238 (83)	
Average	20 (74)	50 (17)	
Bangor Dyslexia			<0.001
Normal	27 (100)	21 (7)	
Moderate	0 (0)	191 (67)	
Severe	0 (0)	76 (26)	
Conners Comprehensive Behavior			0.002
Normal	27 (100)	189 (66)	
Moderate	0 (0)	31 (11)	
Severe	0 (0)	64 (23)	
WJ IV Dyslexia			<0.001
Low Average	0 (0)	266 (92)	
Average	27 (100)	22 (8)	

**Table 3.** Clinical test results.

Biochemical parameters	Control Average ± SD	Cases Average ± SD	*p*-value
Serum Glucose random (Ref < 160 mg/dL)	96.04 ± 16.05	94.01 ± 13.51	0.56
WBCs (Ref 4.0 - 10.0 * 10^9^/L)	7.44 ± 1.16	7.87 ± 1.58	0.12
RBCs (Ref 3.8 - 5.8 * 10^12^/L)	4.69 ± 0.45	4.65 ± 0.51	0.75
Hemoglobin (Ref 12 - 14 g/dL)	12.70 ± 0.70	12.44 ± 0.89	0.11
PVC (Ref 40% - 50%)	42.78 ± 2.41	43.48 ± 4.91	0.30
MCV (Ref 76 - 96 fl)	85.93 ± 6.03	86.09 ± 6.48	0.90
MCH (Ref 27 - 31 pg)	28.59 ± 1.28	28.44 ± 2.22	0.64
MCHC (Ref 32 - 34 g/dL)	32.52 ± 0.80	32.64 ± 1.76	0.60
Platelets (Ref 150 - 400 * 10^9^/L)	301.30 ± 43.15	301.41 ± 53.19	0.99
Polymorph (Ref 40% - 75%)	58.22 ± 8.79	58.45 ± 8.71	0.90
Lymphocytes (Ref 20% - 45%)	32.67 ± 7.31	34.93 ± 7.84	0.17
Monocytes (Ref 01% - 06%)	3.78 ± 1.58	3.73 ± 1.87	0.90
Serum Bilirubin total (Ref < 1.3 mg/dL)	0.84 ± 0.17	0.88 ± 0.95	0.72
Serum ASAT (Ref < 31 U/L)	26.00 ± 5.10	26.25 ± 3.92	0.91
Serum alkaline Phosphate (Ref M/F 100 - 290 U/L, Child: 180 - 615 U/L)	336.60 ± 47.68	329.84 ± 63.23	0.77
Serum creatinine (Ref 0.6 - 1.1 mg/dL)	0.79 ± 0.12	1.12 ± 1.60	0.065
Serum Sodium (Ref 136 - 149 mmol/L)	141.80 ± 3.35	142.01 ± 5.17	0.90
Serum Potassium (Ref 3.2 - 5.2 mmol/L)	4.09 ± 0.47	3.88 ± 0.55	0.38
Serum Chloride (Ref 95 - 105 mmol/L)	98.80 ± 2.77	99.44 ± 3.13	0.64
Hydroxy vit. D3 Level (Ref: Sufficient 30 - 100 ng/ml)	31.40 ± 5.01	20.06 ± 11.97	0.05

## Data Availability

Data are stored and may be available upon reasonable request complying with the current data sharing policy of NIH, available at https://grants.nih.gov/grants/guide/notice-files/NOT-OD-03-032.html. The data sets used in this study include personal information. Thus, datasets in the RAW format are available from SH and MA of COMSTAT University of Pakistan through the corresponding author, SG, on reasonable requests.
